# Nurse delivered focused echocardiography to determine intravascular volume status in a deployed maritime critical care unit

**DOI:** 10.1186/2197-425X-3-S1-A919

**Published:** 2015-10-01

**Authors:** SD Hutchings, L Bisset, L Cantillon, P Keating-Brown, S Jeffreys, C Muzvidziwa, E Richmond, P Rees

**Affiliations:** Kings College Hospital, Critical Care, London, United Kingdom; Royal Centre for Defence Medicine, Birmingham, United Kingdom; Primary Casualty Receiving Facility, Intensive Care Unit, Royal Navy, United Kingdom

## Introduction

Focused echocardiography is increasingly used by clinicians in the management of critically ill patients and has been adopted by the Defence Medical Services as a tool to guide flow assessment and resuscitation in deployed critical care.

## Objectives

We aimed to explore whether two focused echo techniques; Inferior Vena Cava (IVC) and Left Ventricular Outflow Tract (LVOT) Velocity Time Integer (VTi) variability could be taught to a group of critical care nurse who had no previous exposure to ultrasound imaging.

## Methods

Ethical approval was waived for this service improvement study. After a five week program of training validation was carried out on healthy volunteers. The mentor, an accredited focused echo trainer, and six nurses performed a total of 48 scans on 11 volunteers. The mentor and students acquired subcostal long axis and apical five chamber windows using a high frequency linear ultrasound probe (Sonosite M Turbo, P21-51x transducer). Mean values from three measurements were obtained for IVC diameter and LVOT VTi. Minimum and maximum values were recorded for both variables across a full respiratory cycle. Echo images were saved and at least two images for each student were reviewed offline by an accredited echo training supervisor.

## Results

In all cases students were able to obtain adequate echo windows. There was good correlation between values recorded by the mentor and students for both IVC diameter (r = 0.90, p < 0.001) and LVOT VTi (r = 0.77, p < 0.001). Bland Altman analysis showed good correlation with minimal bias for VTi measurements. There was, however, some increase in bias for IVC measurements below 1.2 cm.

## Conclusion

We demonstrated that two focused echo techniques for assessing intravascular volume status could be acquired by specialist nurses, with no previous experience, in a relatively short time and that results were comparable to those produced by an experienced practitioner. These results will need to be replicated in a clinical setting before being adopted into practice.

**Figure 1:**
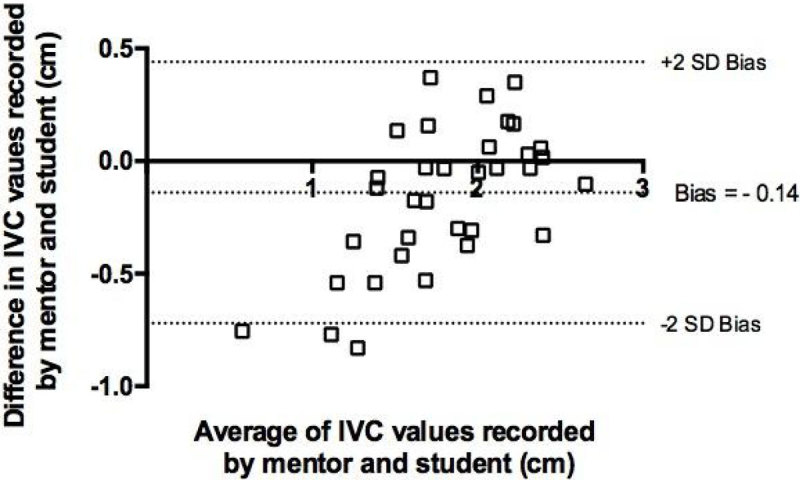
**Bland Altman plot of measured IVC diameters**.

**Figure 2:**
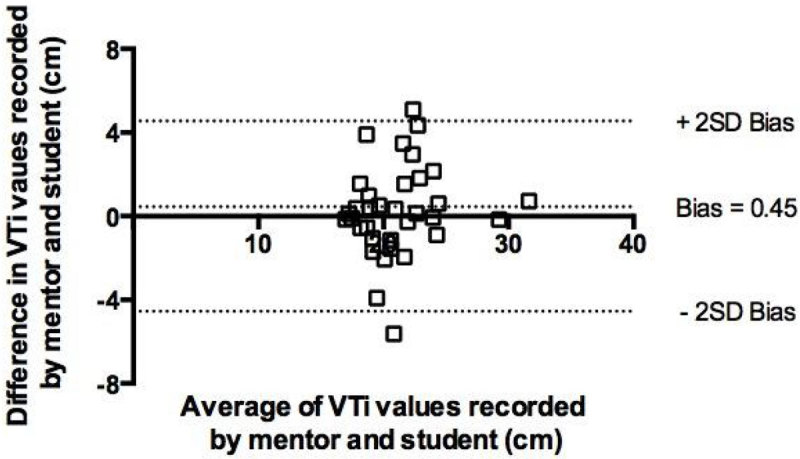
**Bland Altman plot of LVOT VTi measurements**.

